# A yolk sac-derived hepatic γδ T cell subpopulation develops in a hematopoietic stem cell- and thymus-independent manner

**DOI:** 10.1093/procel/pwag037

**Published:** 2026-06-03

**Authors:** Yuzhe Chen, Chaojie Wang, Kangyu Huang, Guoju You, Bing Liu, Yu Lan

**Affiliations:** State Key Laboratory of Experimental Hematology, Institute of Hematology, School of Medicine, Jinan University, Guangzhou 510632, China; State Key Laboratory of Experimental Hematology, National Clinical Research Center for Blood Diseases, Haihe Laboratory of Cell Ecosystem, Institute of Hematology & Blood Diseases Hospital, Chinese Academy of Medical Sciences & Peking Union Medical College, Tianjin 300020, China; State Key Laboratory of Experimental Hematology, Institute of Hematology, School of Medicine, Jinan University, Guangzhou 510632, China; State Key Laboratory of Experimental Hematology, National Clinical Research Center for Blood Diseases, Haihe Laboratory of Cell Ecosystem, Institute of Hematology & Blood Diseases Hospital, Chinese Academy of Medical Sciences & Peking Union Medical College, Tianjin 300020, China; State Key Laboratory of Experimental Hematology, Institute of Hematology, School of Medicine, Jinan University, Guangzhou 510632, China; State Key Laboratory of Experimental Hematology, Haihe Laboratory of Cell Ecosystem, Senior Department of Hematology, Fifth Medical Center, Medical Innovation Research Department, Chinese PLA General Hospital, Beijing 100071, China; State Key Laboratory of Experimental Hematology, Institute of Hematology, School of Medicine, Jinan University, Guangzhou 510632, China; State Key Laboratory of Experimental Hematology, National Clinical Research Center for Blood Diseases, Haihe Laboratory of Cell Ecosystem, Institute of Hematology & Blood Diseases Hospital, Chinese Academy of Medical Sciences & Peking Union Medical College, Tianjin 300020, China; Tianjin Institutes of Health Science, Tianjin 301600, China

**Keywords:** yolk sac endothelial cells, γδ T cells, liver, extrathymic development, hematopoietic stem cell-independent hematopoiesis

## Abstract

γδ T cells are a highly heterogeneous T cell lineage with diverse immunological functions; however, the developmental origins underlying this heterogeneity, particularly the existence and physiological relevance of extrathymic pathways, remain incompletely defined. Our previously generated transcriptomic datasets indicate that *Stab2* is specifically expressed in the yolk sac but not the aortic endothelial cells. Leveraging this spatially restricted expression pattern, we generated a *Stab2-CrexERT2* inducible genetic lineage tracing mouse model and demonstrated its ability to label yolk sac-derived hematopoietic cells *in vivo*. Importantly, we detected the contribution of the yolk sac endothelial cells labeled at embryonic day 9.5 to a subset of Vγ1 γδ T cells in the adult liver but hardly to any hematopoietic progenitors in the thymus and bone marrow. Single-cell transcriptomic profiling combined with functional evaluation revealed that the Stab2-labeled yolk sac endothelial cell-derived hepatic γδ T cells preferentially enrich IFN-γ-producing γδ T1 cells and overrepresent characteristics associated with tissue homeostasis and repair. Collectively, our findings provide direct *in vivo* evidence for the existence of a bone marrow hematopoietic stem cell-independent and thymus-independent developmental pathway of liver γδ T cells originating from yolk sac endothelial cells, and suggest that different origins and developmental paths may confer distinct functional properties to γδ T cells.

## Introduction

γδ T cells are a distinct lineage of T lymphocytes defined by T cell receptors (TCRs) composed of γ and δ chains. These cells exhibit a broad functional spectrum that encompasses both innate and adaptive immune activities, along with diversified mechanisms of antigen recognition and presentation (Hu et al., 2023). Although representing a minor fraction of circulating T cells, γδ T cells play nonredundant roles in tissue surveillance, early immune responses, and maintenance of physiological homeostasis. *Tcrd*-deficient mice display increased epidermal keratinocyte apoptosis and reduced intestinal epithelial turnover, establishing a critical role for γδ T cells in epithelial barrier maintenance (Komano et al., 1995; Sharp et al., 2005). In addition, γδ T cell deficiency has been associated with persistent periodontal inflammation and impaired short-term memory, suggesting broader involvement in tissue homeostasis and neuroimmune regulation (Krishnan et al., 2018; Reisel et al., 2002). By virtue of their MHC-independent recognition and diversified cytotoxic programs, γδ T cells may mitigate tumor immune escape and limit alloreactive complications, including graft-versus-host disease, associated with conventional CAR-T cell therapy. Despite growing appreciation of their functional importance, the developmental origins and lineage diversification of γδ T cells remain incompletely defined.

γδ T cells follow a developmental trajectory distinct from that of αβ T cells. Generally, the thymus serves as the primary site for their development, differentiation, and maturation. Beginning around embryonic day (E) 15.0, the murine fetal thymus generates γδ T cells with distinct molecular and functional characteristics through successive developmental waves. These cells subsequently migrate to peripheral tissues, where they establish long-lived, self-renewing resident populations (Fiala et al., 2020; Ribot et al., 2021). In brief, monoclonal Vγ5Vδ1 γδ T cells emerge around E15.0 and preferentially home to the epidermis; oligoclonal Vγ6Vδ1 and Vγ4 subsets arise several days later; Vγ7 γδ T cells are produced during the perinatal period and predominantly localize to the intestine. Postnatally, polyclonal Vγ1 and Vγ4 γδ T cells are continuously generated from hematopoietic stem cells (HSCs)-derived thymic progenitors and enter the peripheral circulation (Haas et al., 2012). Continuous colonization of the thymus by hematopoietic progenitors is essential for sustained T cell production. Although multi-omics studies indicate that γδ T cells originate from distinct double-negative (DN) progenitors (Sagar, 2024), lineage tracing evidence defining their hematopoietic origins remains limited. Apart from epidermal dendritic epidermal T cells, which derive from embryonic yolk sac hematopoiesis and are maintained through *in situ* self-renewal (Gentek et al., 2018), the developmental origins of most γδ T cell subsets remain poorly characterized.

Emerging evidence also supports the existence of extrathymic developmental pathways for γδ T cells. In nude mice, intestinal γδ T cell populations are largely preserved despite the absence of a functional thymus, suggesting that the intestinal crypt microenvironment can support their local maturation (Bandeira et al., 1991). In addition, transplantation of adult liver hematopoietic progenitor (Lin^−^Sca-1^+^Mac-1^+^, LSM) into thymectomized mice results in the generation of IFN-γ-producing γδ T cells in the liver, implicating extrathymic development for a subset of hepatic γδ T cells (Hu et al., 2021). However, whether these thymus-independent hepatic γδ T cells originate from HSCs or the extraembryonic yolk sac remains unknown. Collectively, due to the limitations of currently available experimental approaches, the development and biological characteristics of extrathymically derived γδ T cells under physiological conditions require further investigation.

The continued refinement of lineage tracing models has demonstrated that multiple adult immune cell populations arise independently of HSCs. These include tissue-resident macrophages—such as microglia in the brain and Kupffer cells in the liver, as well as peritoneal B-1a cells and certain circulating lymphocyte subsets (Hoeffel et al., 2015; Kobayashi et al., 2023). Despite their utility, existing lineage tracing models for studying HSC-independent hematopoiesis (e.g., *Cdh5-CreERT2*, *Csf1r-CreERT2*, and *Runx1-CreERT2*) remain limited by incomplete discrimination between intraembryonic and extraembryonic hematopoietic sources. Single-cell transcriptomic profiling from our laboratory revealed specific expression of *Stab2* in yolk sac endothelial cells (Hou et al., 2022), supporting its potential utility as a marker for yolk sac-derived hematopoiesis. Leveraging this observation, we established an inducible *Stab2-CrexERT2* lineage tracing model to delineate the contribution of yolk sac-derived progenitors to γδ T cell populations across adult tissues.

## Results

### Generation of an inducible *Stab2-CrexERT2* mouse model that specifically labels yolk sac-derived hematopoiesis

By reanalyzing our previously generated single-cell RNA sequencing datasets from E8.0 to E10.0 yolk sac and embryo proper (Hou et al., 2022), we identified the five genes most highly enriched in yolk sac endothelial cells but minimally expressed in embryo proper endothelial populations (Fig. 1A). *Mrc1*, encoding CD206, is predominantly expressed on macrophages, immature dendritic cells, and liver sinusoidal endothelial cells (Sheikh et al., 2000). This expression pattern introduces potential bias and therefore limits its suitability as a marker for unbiased lineage tracing applications. In contrast, *Stab2*, a member of the scavenger receptor family, is predominantly expressed by liver sinusoidal endothelial cells (Schledzewski et al., 2011). Its endothelial-restricted expression pattern makes *Stab2* a suitable candidate for tracing yolk sac-derived hematopoiesis. Accordingly, we generated a *Stab2-CrexERT2* knockin mouse model using a CRISPR/Cas9-mediated gene editing strategy, in which an inducible *CrexERT2* cassette was inserted between exon 69 and the 3′ untranslated region (3′UTR) of the endogenous *Stab2* locus (Fig. 1B). *Stab2-CrexERT2* mice were intercrossed with *CX40-EGFP* arterial reporter mice to enable the identification of arterial endothelial populations. Immunofluorescence staining was conducted to assess estrogen receptor (ER) expression in the yolk sac and embryo proper at E8.5, E9.5, and E10.5. ER expression was evident in yolk sac endothelial cells at E8.5 but was undetectable in the dorsal aorta of the embryo proper at the same developmental stage (Fig. 1C). ER remained undetectable in the dorsal aortic endothelial cells while being robustly expressed in the yolk sac vasculature at E9.5 and E10.5 (Fig. 1D and 1E). Furthermore, ER expression was absent from hematopoietic cells in both the yolk sac and the embryo proper at E8.5, E9.5, and E10.5. Collectively, these data corroborate our transcriptomic results, demonstrating that Stab2 expression is preferentially confined to the yolk sac endothelial cells and excluded from the dorsal aortic endothelial cells where HSCs are generated. Detailed spatial analysis revealed that, at E9.5 and E10.5, ER expression was enriched within the yolk sac venous plexus, with only minor expression detected in arterial endothelial structures (Fig. 1F).

**Figure 1.**
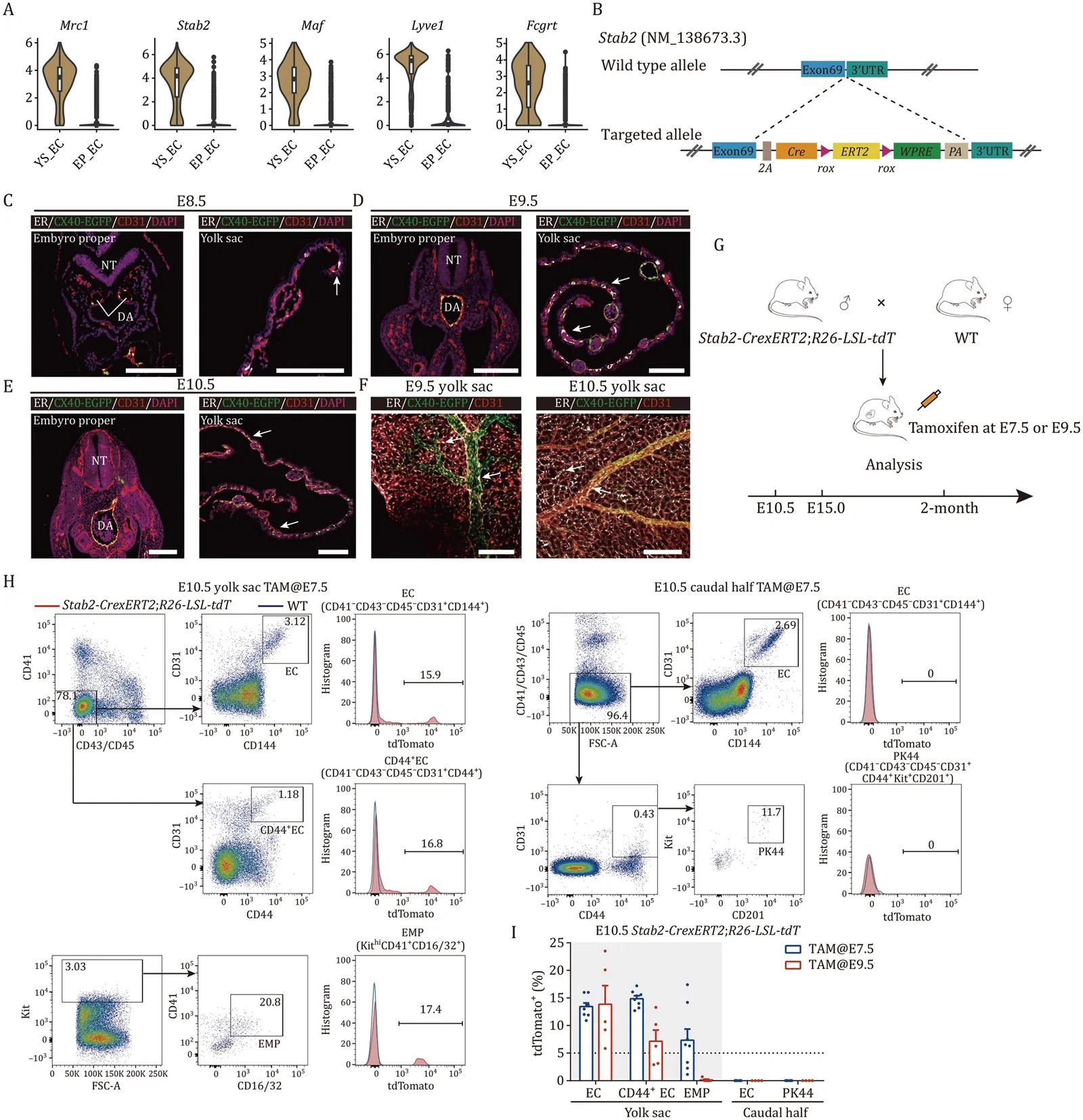
**An inducible *Stab2-CrexERT2* mouse model labels yolk sac-derived hematopoiesis**. (A) Violin plots depicting the top five genes significantly enriched in E8.0–E10.0 yolk sac endothelial cells, with minimal expression detected in embryonic endothelial populations. (B) Schematic representation of the CRISPR/Cas9-mediated gene-targeting strategy used to generate the *Stab2-CrexERT2* lineage tracing mouse line. (C–E) Representative immunofluorescence staining of E8.5 (C), E9.5 (D), and E10.5 (E). AGM and yolk sac sections from *Stab2-CrexERT2*; *CX40-EGFP* embryos. Arrows indicate ER^+^ endothelial cells. Scale bars, 200 μm. (F) Whole-mount immunostaining of E9.5 and E10.5 yolk sac from *Stab2-CrexERT2*; *CX40-EGFP* embryos. The two white lines point to cavities that represent the dorsal aorta. Arrows indicate ER^+^ endothelial cells. (G) Schematic overview of the experimental design for genetic lineage tracing using *Stab2-CrexERT2*; *R26-LSL-tdT* mice. (H) Representative flow cytometry profiling of YS and CH regions from E10.5 *Stab2-CrexERT2*; *R26-LSL-tdT* embryos after temporal induction with tamoxifen at E7.5. (I) Statistical quantification of the flow cytometry data shown in (H). Data are shown as means ± SEM from three independent experiments. The tdTomato^+^ cells below 5% were considered to have negligible contribution. Scale bars, 200 μm. YS: yolk sac, EP: embryo proper, EC: endothelial cell, DA: dorsal aorta, NT: neural tube, CH: caudal half, EMP: erythro-myeloid progenitor.

To determine whether the *Stab2-CrexERT2* specifically fate-maps yolk sac-derived hematopoiesis, we generated *Stab2-CrexERT2*; *R26-LSL-tdT* mice. Wild-type females were crossed with *Stab2-CrexERT2*; *R26-LSL-tdT* males, and temporal recombination was induced by a single dose of tamoxifen administered at E7.5 or E9.5. Embryos were harvested at E10.5, and recombination patterns were analyzed in the yolk sac and in the caudal half region (Fig. 1G). Induction at E7.5 or E9.5 led to robust labeling of yolk sac endothelial cells (CD41^−^CD43^−^CD45^−^CD31^+^CD144^+^), while recombination in endothelial cells of the caudal half region was negligible, confirming the selective expression of Stab2 in extraembryonic yolk sac endothelial cells (Fig. 1H and 1I). Hematopoietic stem and progenitor cells arise in both the yolk sac and the AGM region via endothelial-to-hematopoietic transition (EHT) (Chen et al., 2009; Kissa and Herbomel, 2010). In the AGM, hemogenic endothelium is enriched within the PK44 (CD201^+^CD44^+^Kit^+^) population (Hou et al., 2020, 2024), whereas in the yolk sac, hematopoietic competence progressively consolidates within CD44^+^ endothelial cells from approximately E10.0 (Wang et al., 2021a). Consistent with the overall endothelial labeling pattern, tamoxifen administration at either E7.5 or E9.5 resulted in recombination in a subset of yolk sac CD44^+^ endothelial cells, whereas hemogenic endothelium within the caudal half region was minimally labeled (Fig. 1H and 1I). Notably, induction at E7.5, but not E9.5, led to efficient labeling of the erythro-myeloid progenitors (EMPs) in the yolk sac (Fig. 1H and 1I). This temporal restriction aligns with the known dynamics of yolk sac-derived hematopoiesis. Collectively, these findings demonstrate that temporally controlled activation of the *Stab2-CrexERT2* at E7.5 or E9.5 selectively marks yolk sac-derived hematopoietic output while largely excluding contributions from the caudal half region.

### Adult liver contains yolk sac endothelial cell-derived Vγ1 γδ T cells

To assess the contribution of Stab2-labeled yolk sac endothelial cells to tissue-resident immune cell populations, we administered a single dose of tamoxifen at E7.5 or E9.5 and subsequently traced the contribution to microglia and B-1 cells in 2-month-old mice. Consistent with their early yolk sac origin, microglia (CD45^int^Ly6C^−^CD11b^+^F4/80^+^) exhibited robust labeling upon E7.5 induction, while E9.5 administration resulted in only minimal recombination (Fig. 2A–D). Neither E7.5 nor E9.5 recombination marked B-1 cells in the peritoneal cavity and spleen (Fig. 2B–D), indicating minimal contribution from Stab2-expressing yolk sac endothelial cells to these compartments. γδ T cells across multiple tissues, including the liver, lung, skin, spleen, and lymph nodes, also exhibit tissue-resident properties (Du Halgouet et al., 2024; Qu et al., 2022; Wang et al., 2021b). We next examined γδ T cells in five tissues from 2-month-old mice, including liver, lung, small intestine, spleen, and thymus. Notably, E9.5, but not E7.5 induction labeled a discrete fraction of hepatic γδ T cells (CD45^+^CD3^+^γδTCR^+^), whereas γδ T cells in the lung, small intestine, spleen, and thymus remained unlabeled at both time points (Fig. 2E–D), suggesting a temporally restricted and tissue-specific yolk sac contribution to the liver γδ T cell pool. We next analyzed the Vγ chain usage of this labeled γδ T cell subset and found that the E9.5-labeled γδ T cells predominantly expressed Vγ1 (Fig. 2E and 2F). To define their developmental maturation state, we analyzed CD24 and CD73 expression, established markers of γδ T cell ontogeny (Coffey et al., 2014). TdTomato^+^ cells were enriched within both the CD24^+^CD73^−^ progenitor and CD24^+^CD73^+^ precursor populations, indicating that E9.5-labeled yolk sac-derived cells contribute to early developmental stages of the hepatic γδ T cell compartment (Fig. 2E and 2F).

**Figure 2.**
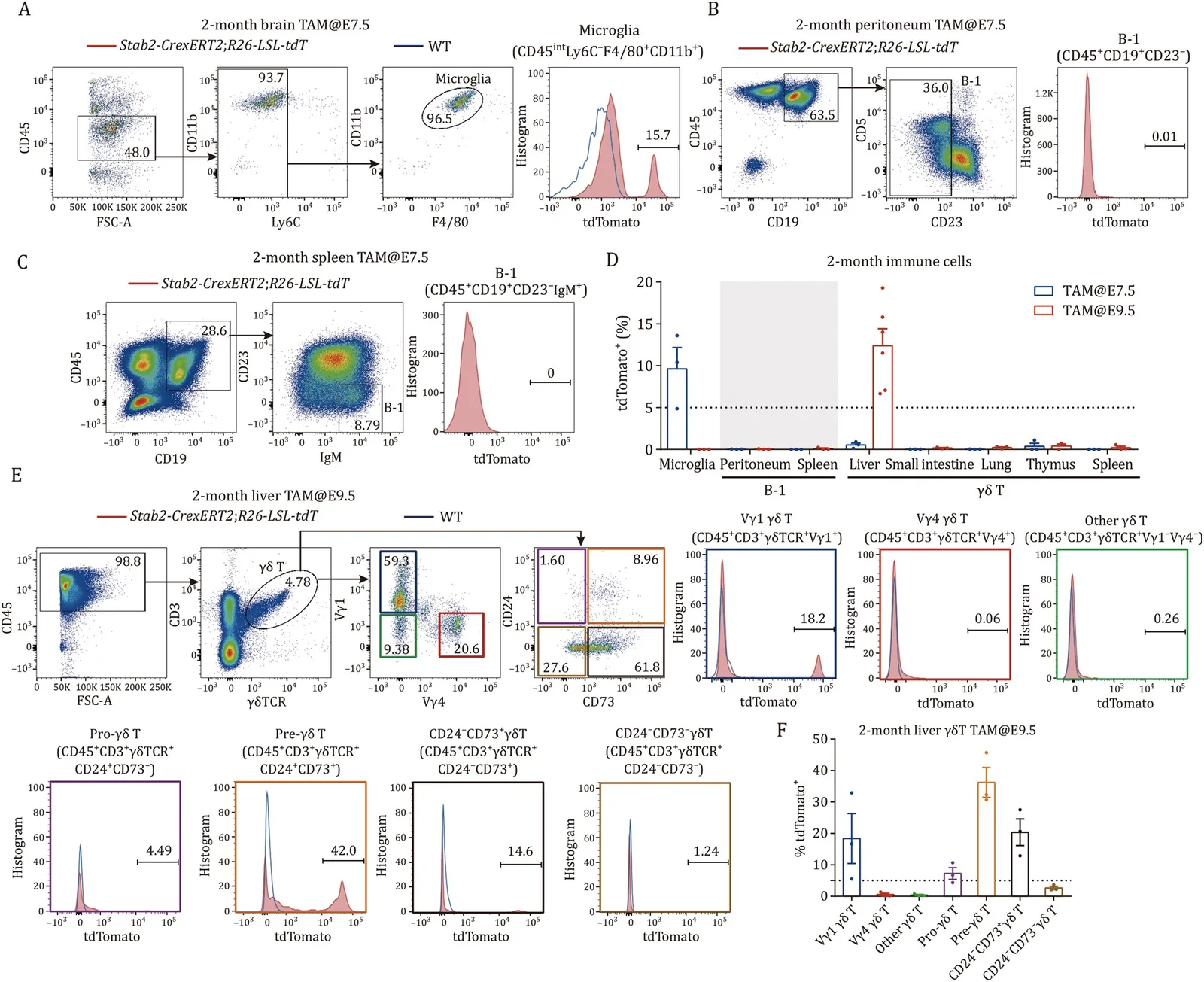
**Yolk sac endothelial cells–derived Vγ1 γδ T cells in the liver**. (A–C) Representative flow cytometric analysis of macrophages (A) and B-1 cells (B and C) in 2-month-old *Stab2-CrexERT2*; *R26-LSL-tdT* mice following tamoxifen induction at E7.5. (E) Representative flow cytometric analysis of γδ T cells in 2-month-old *Stab2-CrexERT2*; *R26-LSL-tdT* mice following tamoxifen induction at E9.5. (D) Statistical quantification of the flow cytometry data shown in (A, B, C, and E). Data are shown as means ± SEM from three independent experiments. (E) Flow cytometric analysis of γδ T cell subsets in 2-month-old *Stab2-CrexERT2*; *R26-LSL-tdT* mice liver following tamoxifen induction at E9.5. (F) Statistical quantification of the flow cytometry data shown in (E). Data are shown as means ± SEM from three independent experiments. The tdTomato^+^ cells below 5% were considered to have negligible contribution.

### E9.5-labeled yolk sac-derived γδ T cells originate in a HSC-independent and thymus-independent manner

To delineate the ontogeny of the E9.5 yolk sac endothelial cell-derived hepatic γδ T cells, we examined the labeling in adult bone marrow hematopoietic stem and progenitor cells, including long-term hematopoietic stem cells (LT-HSCs, LIN^−^Kit^+^Sca-1^+^CD135^−^CD34^−^), short-term hematopoietic stem cells (ST-HSCs, LIN^−^Kit^+^Sca-1^+^CD135^−^CD34^+^), multipotent progenitors (MPPs, LIN^−^Kit^+^Sca-1^+^CD135^+^CD34^+^), common myeloid progenitors (CMPs, LIN^−^Kit^+^Sca-1^−^CD34^+^CD16/32^−^), granulocyte–macrophage progenitors (GMPs, LIN^−^Kit^+^Sca-1^−^CD34^+^CD16/32^+^), and megakaryocyte–erythroid progenitors (MEPs, LIN^−^Kit^+^Sca-1^−^CD34^−^CD16/32^−^) (Cheng et al., 2020). No labeling was detected following E9.5 induction (Fig. 3A), supporting an adult HSC-independent developmental origin.

**Figure 3.**
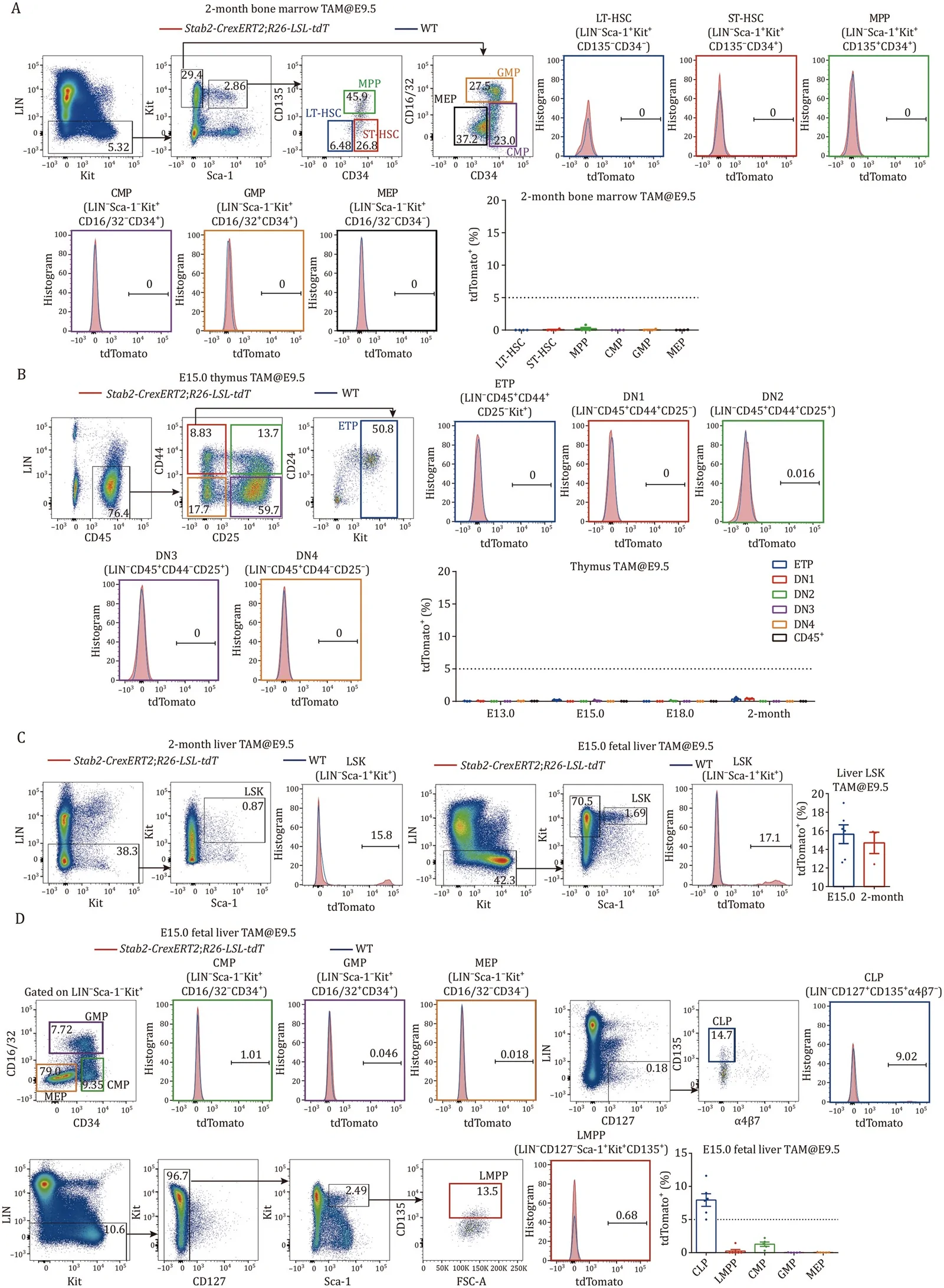
**E9.5 yolk sac-derived γδ T cells originate independent of the thymus**. (A) Representative flow cytometric analysis and statistical quantification of hematopoietic stem and progenitor cells in 2-month-old *Stab2-CrexERT2*; *R26-LSL-tdT* mice bone marrow following tamoxifen induction at E9.5. LIN: TER119, Gr-1, CD11c, CD19, CD127, CD11b, CD3, CD4, CD8α, NK1.1. (B) Representative flow cytometric analysis and statistical quantification of ETP and DNs in embryonic and adult *Stab2-CrexERT2*; *R26-LSL-tdT* mice thymus following tamoxifen induction at E9.5. LIN: TER119, Gr-1, B220, CD4, CD8α, NK1.1. (C) Representative flow cytometric analysis and statistical quantification of LSK (LIN^−^Sca-1^+^Kit^+^) in embryonic and adult *Stab2-CrexERT2*; *R26-LSL-tdT* mice liver following tamoxifen induction at E9.5. LIN: TER119, Gr-1, CD11c, CD19, CD3, NK1.1. (D) Representative flow cytometric analysis and statistical quantification of hematopoietic progenitors in fetal liver following tamoxifen induction at E9.5. LIN (CMP, GMP, and MEP): TER119, Gr-1, CD11c, CD19, CD3, NK1.1. LIN (LMPP, CLP): Ter119, Gr-1, CD11c, CD19, NK1.1, CD3e, CD4, CD8. Data are shown as means ± SEM from three independent experiments. The tdTomato^+^ cells below 5% were considered to have negligible contribution. LT-HSC: Long-term HSC, ST-HSC: Short-term HSC, MPP: multipotent progenitor, CMP: common myeloid progenitor, LMPP: lymphoid-primed multipotent progenitor, CLP: common lymphoid progenitor, MEP: megakaryocyte-erythroid progenitor, GMP: granulocyte-monocyte progenitor, ETP: early thymic progenitor, DN: double negative.

As immature γδ T cells are known to exit in the thymus prior to full maturation and differentiate in peripheral tissues (Joannou et al., 2022), we evaluated the labeling in thymic progenitor compartments across embryonic (E12.0, E15.0, and E18.0) and adult (2-month) stages. E9.5 induction failed to label ETPs (LIN^−^CD45^+^CD44^+^CD25^−^Kit^+^), any DN subsets, or CD45^+^ cells at all time points analyzed (Fig. 3B), providing compelling evidence that the E9.5 yolk sac-derived γδ T cells develop independent of the thymus.

The robust labeling of immature γδ T cells in the adult liver suggested that the liver may serve as a developmental niche for this subset. The fetal liver serves as the principal hematopoietic organ during murine embryogenesis, and mouse adult liver also contains hematopoietic stem cells derived from the fetal liver (Bai et al., 2021). We further examined LSK (LIN^−^Sca-1^+^Kit^+^) compartments, which enrich hematopoietic stem progenitor cells, in 2-month-old mice liver and fetal liver. E9.5 induction resulted in the labeling of LSK in both fetal and adult liver (Fig. 3C). Furthermore, E9.5 induction selectively labeled the common lymphoid progenitors (CLPs, LIN^−^CD127^+^CD135^+^α4β7^−^) population in the E15.0 fetal liver, whereas other progenitor subsets, including lymphoid-primed multipotent progenitors (LMPPs, LIN^−^CD127^−^Kit^+^Sca-1^+^CD135^+^), CMPs, GMPs, and MEPs, remained unlabeled (Fig. 3D). This selective labeling suggests the emergence of a yolk sac-derived progenitor population with lymphoid-restricted potential in the fetal liver, which may be retained within the hepatic niche and serve as a direct source of hepatic γδ T cells.

### Characterizing E9.5 yolk sac-derived γδ T cells by single-cell transcriptomic analysis

To delineate the molecular features of E9.5-labeled γδ T cells, we conducted single-cell RNA sequencing on tdTomato^+^ cells in 2-month-old mice liver isolated by flow cytometry. This population encompassed all γδ T cells and a minor proportion of other cell types, with tdTomato^−^ counterparts analyzed in parallel as controls (Fig. 4A). Unsupervised clustering resolved 13 transcriptionally distinct cell populations in the liver, which were further refined and annotated according to their characteristic gene expression profiles, including natural killer cells (*Ncr1, Klrd1*), innate lymphoid cells (*Il7r, Gata3*), αβ T cells (*Cd3e, Trac*), γδ T cells (*Cd3e, Trdc*), B cells (*Cd79a, Cd19*), dendritic cells (*H2-Aa, Irf8*), monocytes (*Lyz2, Ctss*), macrophages (*Cd68, C1qa*), neutrophils (*S100a8, S100a9*), endothelial cells (*Kdr, Pecam1*), mesenchymal cells (*Dcn, Col3a1*), hepatocytes (*Apoc3, Ttr*), and cholangiocytes (*Cldn3, Spp1*) (Fig. 4B and 4C; Table S1). Notably, six γδ T cell populations were identified, characterized by the expression of *Trdc*, *Trgc1*, *Trgv1*, and distinct TCR repertoires (Fig. 4C and 4D). We next focused on the γδ T cell populations to investigate their molecular characteristics and developmental dynamics (Fig. 4E–G; Table S2). The gdT_Sell cluster corresponds to a naïve γδ T cell population in the liver, defined by elevated expression of migration-associated genes (*Sell* and *S1pr1*) and maturation-related transcription factor genes (*Tcf7* and *Lef1*) (Arbones et al., 1994; Okamura et al., 1998). The gdT_Gzmk, gdT_Il4, and gdT_Ncr1 clusters both exhibited high expression of *Ifng*, *Cd27*, and *Il2rb*, suggesting that they represent IFN-γ-producing γδ T1 cells (Hu et al., 2021; Zhong and Zhu, 2017). Additionally, gdT_Gzmk displayed elevated levels of *Casp1* and *Gzmk*, consistent with a pro-inflammatory phenotype, whereas gdT_Ncr1 and gdT_Il4 were enriched for *Il4*, *Fcer1g*, *Tyrobp*, and *Klre1*, indicative of an NK-like functional program. The gdT_Rorc cluster represents γδ T17 cells, characterized by high expression of *Rorc*, *Ccr6*, *Itgae*, and *Il17a* (Hu et al., 2021; Ivanov et al., 2006). The gdT_Prolif cluster exhibited elevated expression of proliferation-associated genes, including *Mki67*, *Tpx2*, and *Cdk1* (Fig. 4F and 4G). Supportively, the expression pattern of several known transcriptional regulators in the identified γδ T subsets is consistent with established γδ T cell differentiation programs (Pinheiro and Silva-Santos, 2025) (Fig. 4H). Correlation analysis of tdTomato^+^ and tdTomato^−^ cells showed that same annotated γδ T cell populations essentially clustered together, indicating the largely preserved γδ T cell identities regardless of tdTomato labeling (Fig. 4I). Notably, gdT_Sell and gdT_Gzmk subsets were enriched in tdTomato^+^ γδ T cell clusters (Fig. 4J), in line with the results that E9.5 induction sufficiently labeled early developmental stages of the hepatic γδ T cell compartments.

**Figure 4.**
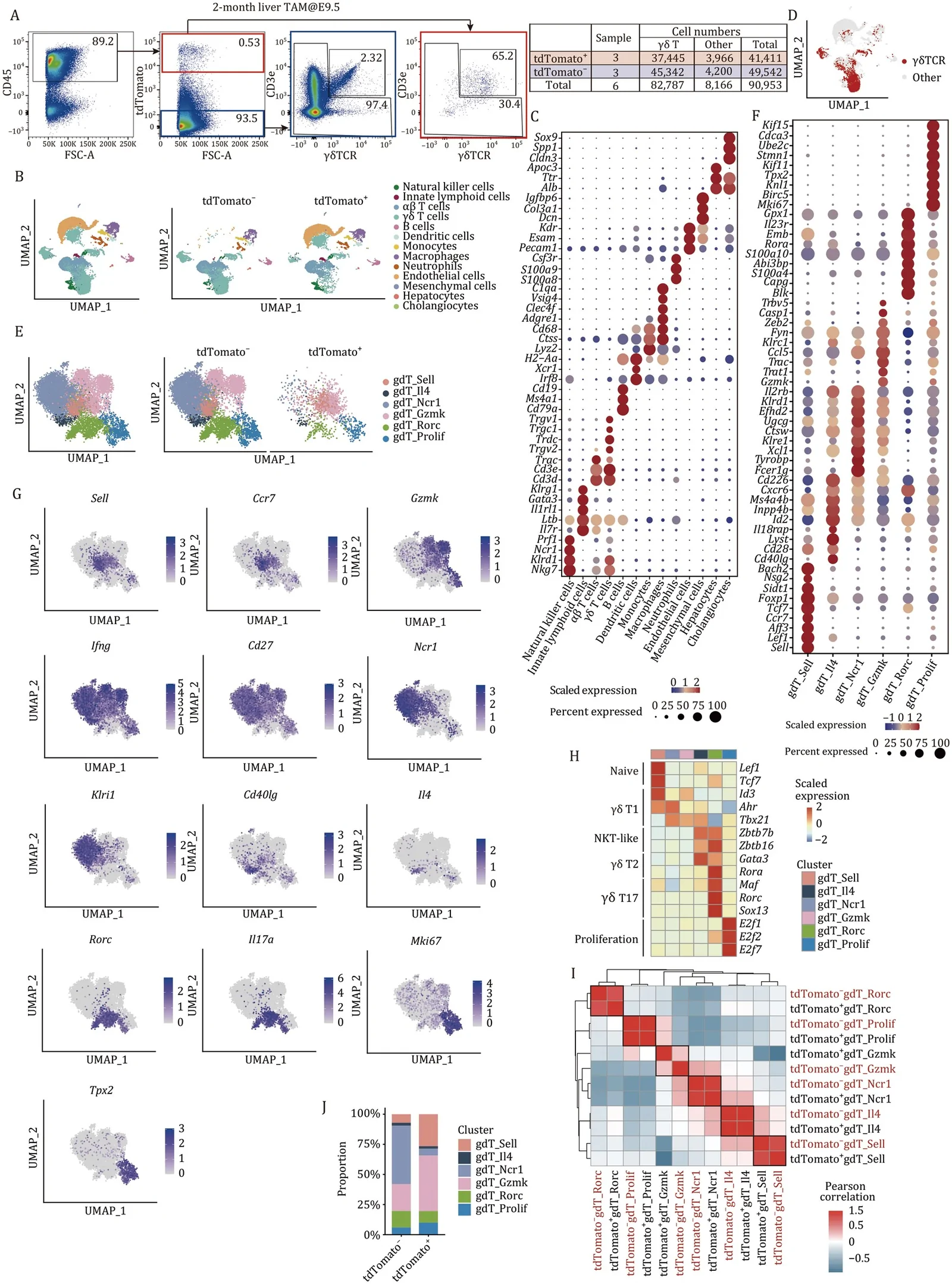
**Features of E9.5 yolk sac-derived γδ T cells based on single-cell transcriptomic analysis**. (A) Flow cytometry gating strategy for single-cell RNA sequencing in 2-month-old *Stab2-CrexERT2*; *R26-LSL-tdT* mice liver following tamoxifen induction at E9.5. The table displaying FACS-purified cell information. (B) Visualization of Uniform Manifold Approximation and Projection (UMAP) for liver cells from 2-month-old mice, with cells colored by types. (C) Dot plot showing the scaled expression of signature genes in each cell cluster. (D) UMAP showing TCR repertoire distribution in hepatic cells, as defined in panel B. (E) UMAP visualization of distinct subclusters of hepatic γδ T cells. (F) Dot plot showing the scaled expression of differentially expressed genes in each γδ T subset. (G) UMAP plot showing the expression of signature genes for γδ T subsets. (H) Heatmap showing the scaled expression of representative transcription factor genes across hepatic γδ T cell clusters. (I) Heatmap showing correlation analysis of tdTomato^+^ and tdTomato^−^ cells within each γδ T cell cluster based on principal component analysis embeddings. (J) Bar plot presenting the proportion of subclusters with tdTomato^−^ and tdTomato^+^ γδ T cells.

Our lineage tracing data suggested that, among the liver γδ T cell populations we identified, the yolk sac-derived tdTomato^+^ subset is less likely to originate from the thymus. To further compare the molecular features of the γδ T cells with different origins, we used published single-cell datasets of thymic and hepatic γδ T cells from 2-month-old mice as a reference dataset (Hu et al., 2021) and integrated them with our own dataset (Fig. 5A–C). The published thymic and hepatic γδT cells were reannotated as naive (*Sell* and *S1pr1*), T1-like (*Ifng* and *Klrb1c*), T17-like (*Il17a* and *Rorc*), and proliferating (*Mki67* and *Stmn1*) sub-clusters (Fig. 5A and 5B). As expected, integrative analysis showed that our gdT_Sell aligned with naive γδT cells in the reference dataset; gdT_Gzmk/gdT_Il4/gdT_Ncr1 with γδ T1; gdT_Rorc with γδ T17; and gdT_Prolif with the corresponding proliferating population, respectively (Fig. 5D). Notably, tdTomato^-^ but not tdTomato^+^ naïve γδ T cells clustered in closer proximity to thymic naïve γδ T cells on UMAP plot (Fig. 5D). This relationship was supported by transcriptomic similarity, showing that tdTomato^−^ naïve γδ T cells are more closely aligned with thymic naïve γδ T cells (Fig. 5E and 5F). We next compared gene expression profiles between naïve γδ T cell clusters from the thymus and liver (Fig. 5G; Table S3). Proliferation-associated genes, including *Cd24a* and *Sox4*, were more highly expressed in the thymic naïve γδ T cell cluster, suggesting an earlier developmental stage, consistent with previous reports (Hu et al., 2021). In contrast, the tdTomato^+^ hepatic naïve γδ T cell cluster was enriched for genes associated with tissue residency and functional priming, including *Ehd3*, *Igfbp7*, *Ifitm3*, *Fcgr2b*, *Dlc1*, suggesting a bias toward differentiation into IFN-γ-producing γδ T1 cells and enhanced granzyme-mediated activity (Kumar et al., 2025; Sun et al., 2024). Consistent with the computational prediction, intracellular cytokine staining following *ex vivo* PMA and ionomycin stimulation showed that tdTomato^+^ hepatic γδ T cells produced significantly more IFN‑γ and less IL‑17 than tdTomato^−^ counterparts (Fig. 5H and 5I). Collectively, these findings indicate that Stab2‑labeled yolk sac‑derived hepatic γδ T cells are biased toward the IFN‑γ‑producing γδ T1 cells.

**Figure 5.**
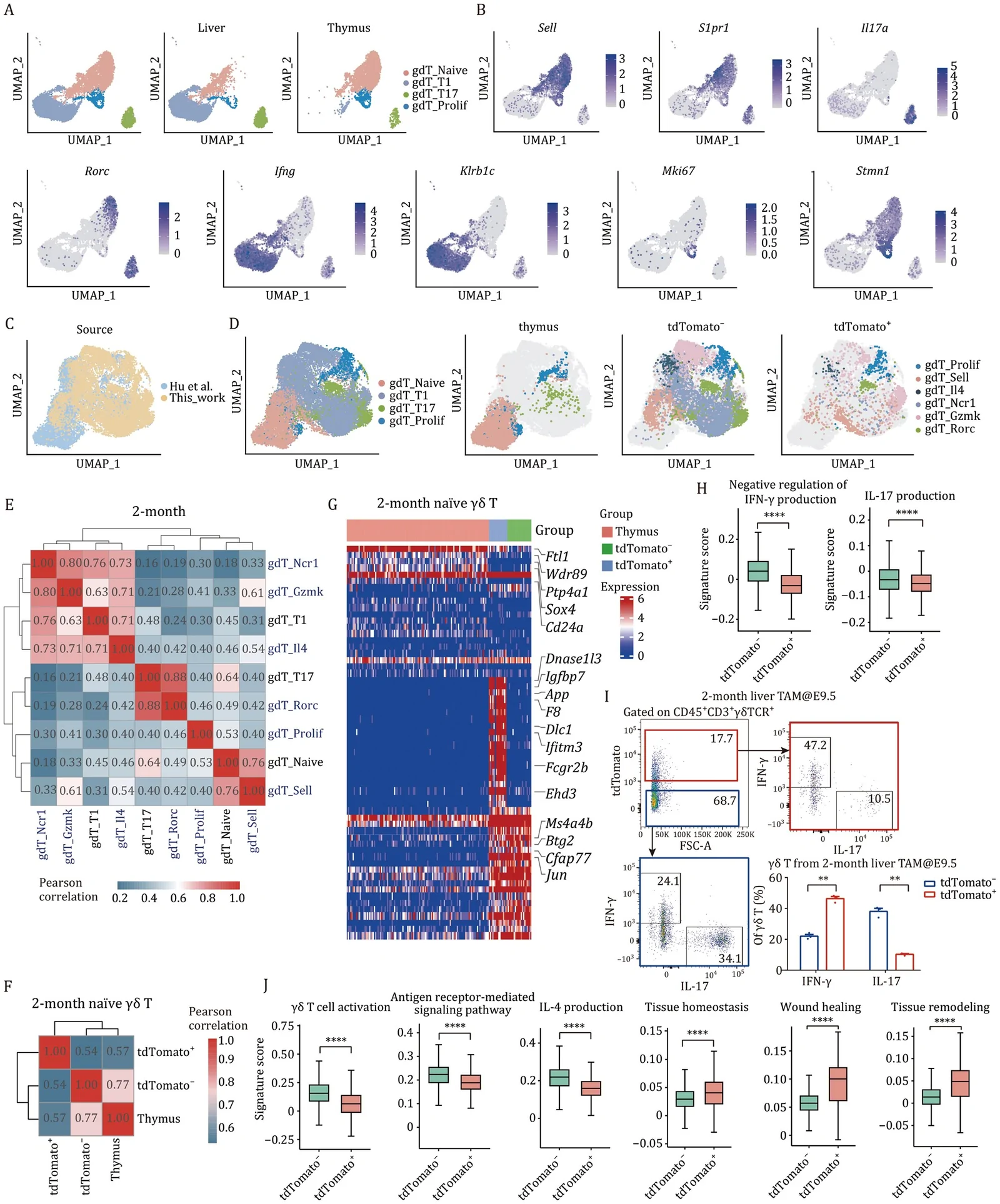
**Comparative analysis of the γδ T cells with distinct origins**. (A) UMAP plot showing hepatic and thymic γδ T subclusters from 2-month-old mice, with cells colored by different sources. Public data was downloaded from GSE164106. (B) UMAP plot showing the expression of signature genes for each cell type, as defined in panel A. (C) UMAP representing integrated γδ T subclusters from public and in-house data, with cells colored by datasets. (D) UMAP plot showing γδ T subclusters from different sources. (E) Heatmap shows the correlation across different γδ T clusters. (F) Heatmap showing the correlation among thymic, tdTomato^-^, and tdTomato^+^ naïve γδ T cell populations. (G) Heatmap showing the expression of differentially expressed genes among thymic, tdTomato^−^, and tdTomato^+^ naïve γδ T cell populations. (H) Box plots showing Gene Ontology (GO) signature analysis of IFN-γ and IL-17 production signaling in tdTomato^+^ and tdTomato^−^ hepatic γδ T cells from 2-month-old mice (****P < 0.0001). (I) Representative flow cytometry plots and quantification of IFN-γ and IL-17 expression by tdTomato^+^ and tdTomato^−^ liver γδ T cells from 2-month-old *Stab2-CrexERT2*; *R26-LSL-tdT* mice treated with tamoxifen at E9.5. tdTomato^+^ γδ T cells show significantly higher IFN-γ and lower IL-17 production compared to their tdTomato^−^ counterparts (**P < 0.01, *n *= 3). (J) Box plots showing GO signature analysis of representative biological processes in tdTomato^+^ and tdTomato^−^ hepatic γδ T cells from 2-month-old mice (****P < 0.0001).

Previous studies have shown that murine Vγ1^+^ γδ T cells exhibit diverse context-dependent functions. They participate in microbial antigen recognition and phosphoantigen sensing (Born et al., 2013; Happ et al., 1989), supporting their involvement in early immune surveillance. Furthermore, they are implicated in the interleukin-4-mediated negative regulation of antitumor immunity (Hao et al., 2011), the promotion of airway inflammation (Hahn et al., 2004), and the tissue-protective responses in brain injury (Abou-El-Hassan et al., 2023) and myocarditis (Huber and Sartini, 2005). By comparison, the tdTomato^−^ γδ T cells in adult liver showed enrichment of γδ T cell activation, antigen receptor-mediated signaling pathway, and interleukin-4 production, whereas the tdTomato^+^ counterparts were characterized by higher scores for tissue homeostasis, wound healing, and tissue remodeling (Fig. 5J). These characteristics are consistent with previously reported features of yolk sac-derived macrophages (Elhag et al., 2021). Moreover, yolk sac-derived dendritic epidermal T cells have also been reported to exhibit tissue repair capacity (Gentek et al., 2018). These findings collectively suggest that yolk sac-derived immune cells possess a low pro-inflammatory and high repair-oriented functional propensity, thereby might playing a distinctive role in adult tissue homeostasis and injury repair.

Taken together, this study revealed the existence—under physiological conditions—of a distinct developmental pathway for a subset of γδ T cells, which originate in the extraembryonic yolk sac, develop in the liver, and bypassing both hematopoietic stem cells and the thymus. These yolk sac-derived hepatic Vγ1 γδ T cells preferentially enrich γδ T1-like subset, showing relatively high capacity to produce IFN-γ (Fig. 6).

**Figure 6.**
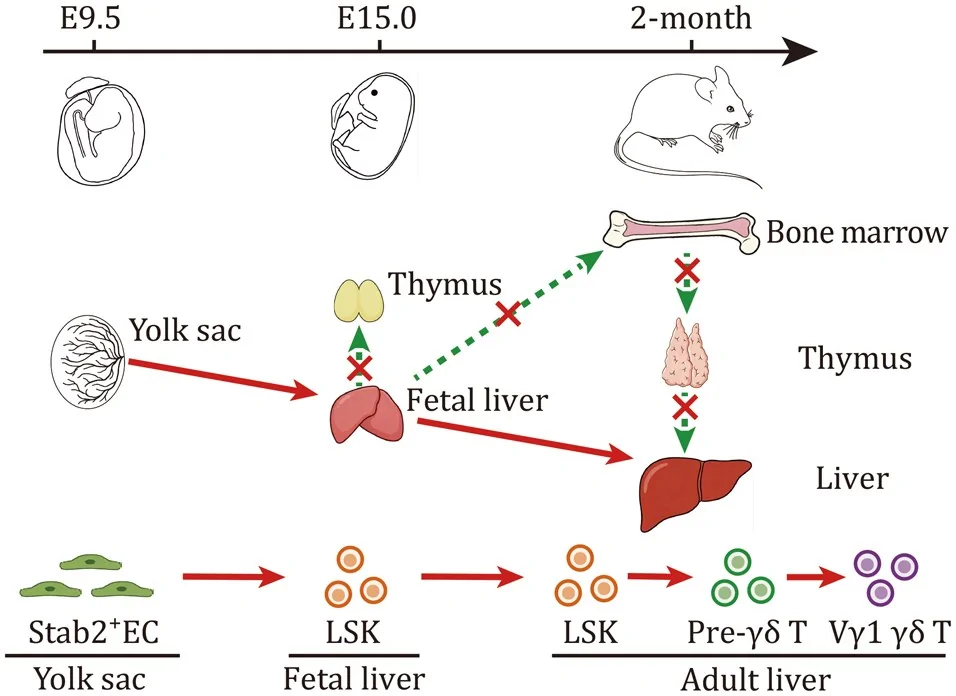
**Schematic diagram summarizing the development pathway of the yolk sac-derived hepatic γδ T cell subpopulation**. Solid arrows indicate the suggested developmental pathway revealed in this study. Dashed arrows indicate the classic pathways for hematopoietic stem and progenitor cell colonization and T cell development, which is not the manner for the development of the yolk sac-derived specific subtype of hepatic γδ T cells. EC, endothelial cells; LSK, Lineage^−^Sca-1^+^Kit^+^ hematopoietic stem progenitor cells.

## Discussion

A major obstacle in developmental hematology has been the lack of tools to definitively resolve the site origin and long-term contribution of successive hematopoietic waves *in vivo*, thereby obscuring their presumed distinct functional roles in embryogenesis and adult tissue homeostasis. Here, we establish a *Stab2-CrexERT2* inducible genetic lineage tracing mouse model that enables the specific fate mapping of yolk sac-derived hematopoiesis *in vivo*. Temporal induction at E7.5 or E9.5 preferentially targets yolk sac endothelial cells without marking aortic endothelial cells, allowing the *Stab2-CrexERT2* to dissect yolk sac-derived hematopoiesis despite the partially overlapping time windows of different hematopoietic waves. Furthermore, Stab2-labeled yolk sac endothelial cells do not contribute to bone marrow HSCs, supporting its utility as a tool for investigating HSC-independent hematopoiesis in adult mice. This refined specificity substantially improves the *in vivo* resolution of hematopoietic lineage tracing.

γδ T cells were initially identified in 1984 (Saito et al., 1984), and subsequent studies have established them as a distinct T cell lineage with potent immunoregulatory and cytotoxic functions, positioning them as promising targets for immunotherapeutic intervention. Canonically, γδ T cells are thought to arise in the thymus, where strong TCR signaling in double-negative thymocytes instructs commitment to the γδ lineage (Munoz-Ruiz et al., 2017). While the existence of extrathymic γδ T cells in the intestine and liver has been described, the supporting evidence primarily stems from thymectomy models. Consequently, these observations may be confounded by compensatory adaptations to thymic removal and may not accurately represent γδ T cell development under steady-state physiological conditions. By employing a newly established inducible genetic lineage tracing mouse model that permits physiological scenario-based tracking of the yolk sac-derived progeny, we uncover a distinct population of hepatic γδ T cells that originates from the yolk sac and develops independently of the thymus.

Collectively, our findings refine current understanding of extra-thymic γδ T cell ontogeny and establish a foundation for systematically interrogating the origin-dependent functional specialization of γδ T cells. Our research has opened up new directions for future studies, including the upstream transcriptional circuitry and signaling networks orchestrating the ontogeny and hepatic residency of this extra-thymic γδ T cell subset, and the specific functions and mechanisms of the yolk sac-derived hepatic γδ T cells in homeostasis and diseases. These studies will be of profound significance for understanding the functional diversification of immune cells.

## Supplementary Material

pwag037_Supplementary_Data

## Data Availability

All raw and processed data underlying this article have been deposited in the Gene Expression Omnibus (GEO) database under accession number GSE329257. Public datasets of hepatic and thymic γδ T cells were obtained from GSE164106 (Hu et al., 2023).

## Code availability

This paper does not report original code. Any additional information required to reanalyze the data reported in this paper is available from the lead contact upon request.

## Artificial intelligence (AI)

No AI-assisted technologies were used in the preparation of this manuscript.

## Abbreviations

AGM: aorta–gonad–mesonephros
CLP: common lymphoid progenitor
CMP: common myeloid progenitor
DN: double-negative
E: embryonic day
EMP: erythro-myeloid progenitor
ER: estrogen receptor
GMP: granulocyte–macrophage progenitor
HSC: hematopoietic stem cell
LMPP: lymphoid-primed multipotent progenitor
MEP: megakaryocyte–erythroid progenitor
MPP: multipotent progenitor
TCR: T cell receptor
