# Supplementary material for: A yolk sac-derived hepatic γδ T cell subpopulation develops in a hematopoietic stem cell- and thymus-independent manner
Source: Protein Cell. 2026 Jun 3;17(10):922–33. doi: 10.1093/procel/pwag037 (PMC13626739; doi:10.1093/procel/pwag037)
Supplement: Supplementary file 1 [file pwag037_supplementary_data.zip › PAC-26323-LY-Supplemental Materials.pdf]

Supplemental Materials

A yolk sac-derived hepatic γδ T cell subpopulation develops in a hematopoietic stem cell- and thymus-independent manner

Yuzhe Chen[1,4], Chaojie Wang[2,4], Kangyu Huang[1,4], Guoju You[2,4], Bing Liu[1,3,*] (ORCID: 0000-0002-7313-6924), Yu Lan[1,2,*] (ORCID: 0000-0002-4731-5945)

This file includes Methods with corresponding references and Supplemental table legends.

Methods

Mice

Mice were housed at the Laboratory Animal Center of the Academy of Military Medical Sciences under specific pathogen-free conditions with a 12-hour light/12-hour dark cycle. All animal procedures were approved by the Institutional Animal Care and Use Committee (IACUC) and conducted in strict accordance with relevant institutional and national guidelines. *Rosa26*$^{LoxP\text{-}Stop\text{-}LoxP\text{-}tdTomato}$ mice were described previously **(Madisen et al., 2010).** *Stab2-CrexERT2* mouse line was generated using CRISPR/Cas9-mediated knock-in technology by the Shanghai Model Organisms Center. We crossed these two lines to generate the *Stab2-CrexERT2;Rosa26*$^{LoxP\text{-}Stop\text{-}LoxP\text{-}tdTomato}$ mice (referred to as *Stab2-CrexERT2;R26-LSL-tdT*) for genetic lineage tracing studies. All mouse strains were maintained on a C57BL/6 genetic background. Embryonic stages were determined based on somite pair (sp) counts as follows: E8.5, 8–12 sp; E9.5, 21–30 sp; and E10.5, 36–40 sp. For lineage tracing experiments, tamoxifen (Sigma, T5648) was dissolved in corn oil and administered to pregnant females by oral gavage at E7.5 or E9.5 at a dosage of 0.1 mg per gram of body weight.

Single cell preparation

Embryonic tissues

Yolk sac, caudal half, and fetal thymus tissues were enzymatically digested in 500 μL of buffer containing 0.01 mg/mL collagenase I, 0.05 mg/mL DNase I, and 20% fetal bovine serum (FBS) in phosphate-buffered saline (PBS) at 37°C for 30 minutes with gentle agitation.

Fetal liver tissues were mechanically dissociated using a syringe fitted with a 0.45-mm inner diameter needle, followed by filtration through a 40-μm cell strainer to generate single-cell suspensions.

Adult tissues

All animals were euthanized by cervical dislocation and subsequently perfused with 10 mL

PBS to remove circulating blood cells.

Brain, liver, lung and small intestine were collected. Single-cell suspensions were obtained as previously described **(Liu et al., 2020).** Briefly, tissues were minced into small fragments and incubated in 5 mL digestion buffer containing 0.2 mg/mL collagenase IV, 0.05 mg/mL DNase I, and 10% FBS in RPMI 1640 medium. Samples were digested at 37°C for 60 minutes with gentle agitation. Following enzymatic digestion, tissues were mechanically dissociated using a syringe fitted with a 1.2-mm inner diameter needle and passed through a 70-μm cell strainer. Cell suspensions were centrifuged at 380 x g for 5 minutes, and the resulting cell pellet was resuspended in 8 mL of 40% Percoll solution. Subsequently, 5 mL of 80% Percoll solution was carefully underlaid to establish a discontinuous density gradient. Samples were centrifuged at 1,580 x g for 20 minutes at room temperature with acceleration 1 and no brake. Mononuclear cells were collected from the interphase layer for further analysis.

For thymus and spleen, tissues were mechanically dissociated by passing them through 40-μm cell strainers into sterile PBS to generate single-cell suspensions.

Flow cytometry

Cells were analyzed and sorted by flow cytometers Fortessa and FACS Aria III (BD Biosciences). The following antibodies were used: TCRγδ-BV605 (Biolegend, GL3), CD3e-APC-Cy7 (eBioscience, 145-2C11), CD45-FITC (eBioscience, 30-F11), CD24-BV510 (Biolegend, M1/69), CD73-APC (Biolegend, TY/11.8), TCR Vγ1.1 (Biolegend, 2.11), TCR Vγ2 (Biolegend, UC3-10A6), CD45-BV421 (BD, 30-F11), F4/80-APC-Cy7 (Biolegend, BM8), CD11b-FITC (eBioscience, M1/70), Ly6C-BV786 (Biolegend, HK1.4), Ly6G-PE-Cy7 (Biolegend, 1A8), CD19-BV510 (eBioscience, eBio1D3), CD5-BV421 (Biolegend, 53-7.3), CD23-APC-Cy7 (Biolegend, B3B4), CD41-PE-Cy7 (eBioscience, eBioMWReg30), CD43-PE-Cy7 (BD, MWReg30), CD201-APC (eBioscience, eBio1560), CD144-BV421 (Biolegend, BV13), CD31-BV786 (BD, MEC13.3), Kit-APC-Cy7 (eBioscience, 2B8), CD44-APC-Cy7

(Biolegend, IM7), CD16/32-BV510 (Biolegend, 93), Kit-BV650 (Biolegend, ACK2), CD25-PE-Cy7 (eBioscience, PC61.5), CD34-BV421 (eBioscience, RAM34), Sca-1-FITC (eBioscience, D7), CD127-PE-Cy7 (eBioscience, A7R34), α4β7-APC (Biolegend, DATK32), CD135-BV421 (Biolegend, A2F10), TER119-Biotin (Biolegend, TER119), Gr-1-Biotin (Biolegend, RB6-8C5), CD11c-Biotin (Biolegend, N418), CD4-Biotin (eBioscience, GK1.5), CD8α-Biotin (eBioscience, 53-6.7), NK1.1-Biotin (eBioscience, PK136), Streptavidin-BV421 (eBioscience). Data were analyzed using FlowJo v10.8.1 (Treestar) and Prism 6 (GraphPad) softwares.

Immunofluorescence

Immunofluorescence staining of embryo was performed as previously described **(Hou et al., 2022; Tang et al., 2021)**. Briefly, embryos were fixed, paraffin-embedded, and sectioned into 5-μm slices. After deparaffinization and rehydration, tissue sections were incubated in 3% hydrogen peroxide (H_2O_2) for 30 minutes at room temperature to quench endogenous peroxidase activity. Sections were then blocked for 30 minutes using blocking solution (Zhongshan Golden Bridge, ZLI-9056) to reduce nonspecific binding. Following blocking, sections were incubated overnight at 4°C with the following primary antibodies: anti–Estrogen Receptor (ER) (Abcam, ab16660, 1:200), anti-EGFP (Cell Signaling Technology, 2956S, 1:200), or anti-CD31 (Cell Signaling Technology, 77699S, 1:100). After washing, sections were incubated with the appropriate secondary antibodies (Zhongshan Golden Bridge) for 30 minutes at room temperature. Sections were then stained with Dendron Fluor TSA (Histova, NEON 5-color IHC Kit for FFPE, NEFP450, 1:75) for 1 min. Finally, sections were stained in DAPI (Histova, 1:500) for 5 minutes at room temperature.

Single-cell RNA-seq and data processing

For 10X Genomics-based scRNA-seq, we implemented the Chromium Single Cell 5’ PE v3 libraries according to the manufacturer’s instructions (https://cdn.10xgenomics.com/image/upload/v1710231087/support-documents/CG000733_ChromiumGEM-

X_SingleCell5_ReagentKitsv3_UserGuide_RevA.pdf). Sequencing was performed on the NovaSeq X PLUS pe150 platform. Raw files were processed with Cell Ranger (version 10.0.0) pipeline coupled with mouse reference GRCm39-2024-A. 10x Chromium Single-Cell V(D)J reference was set as refdata-cellranger-vdj-GRCh38-alts-ensembl-7.0.0. The output gene expression matrices were analyzed by R software (version 4.4.1) with the Seurat package (version 4.4.0). Low-quality cells were removed if they met any of the following criteria: (1) < 800 unique molecular identifiers (UMIs); (2) < 500 genes; or (3) > 10% UMIs mapped on the mitochondrial genome. Doublets were identified by the R package DoubletFinder (v.2.0.3) **(McGinnis et al., 2019)** with default settings. After the removal of the low-quality cells and doublets, a total of 26,972 cells and 33,696 genes were kept for further analysis.

Data integration and cell-type annotation

For the integrative analysis of published thymic and hepatic γδ T cells from 2-month-old mice with in-house data, we performed integration analysis via the FastMNN method from the SeuratWrappers package (v.0.3.1) **(Haghverdi et al., 2018)**. The integrated datasets were subsequently subjected to dimensionality reduction and downstream analyses. Each cluster was manually assigned to a most likely cell type based on the canonical cell-type markers.

Differential gene expression

Differentially expressed genes were recognized using the “FindAllMarkers” function in Seurat (Wilcoxon rank-sum test, with p-value adjusted for multiple testing using Bonferroni correction). Genes with adjusted *P* value less than 0.05 were considered as significant.

Gene set scoring analysis

Gene set scoring analysis for tdTomato^{+} and tdTomato^{-} γδ T cells was performed using “AddModuleScore” function in Seurat. Gene sets subjected to scoring analysis included “negative regulation of type II interferon production” (GO:0032689), “interleukin-17 production” (GO:0032620),“gamma-delta T cell activation” (GO:0046629), “antigen receptor-mediated

signaling pathway" (GO:0050851), "interleukin-4 production" (GO:0032633), "tissue homeostasis" (GO:0001894), wound healing (GO:0009611) and "tissue remodeling" (GO:0048771) .

Regression of endothelial-associated features

To mitigate the confounding effect of endothelial-associated transcriptional features in γδ T-cell analysis, an endothelial-associated gene program from differential expression analysis across unsupervised clusters. Specifically, the top 100 genes enriched in the subcluster with endothelial-like features were used as endothelial signatures, and module scores were computed by Seurat's function "AddModuleScore". Principal component analysis (PCA) was performed on variable features after excluding the endothelial signature genes. The correlation between endothelial signature score and each principal component was assessed by Pearson correlation analysis. Principal components with correlation coefficients below 0.15 were retained for downstream neighbor graph construction, dimensionality reduction, and clustering.

Correlation analysis

To reveal similarity of γδ T cells from different groups, γδ T cells were integrated for Pearson correlation analysis based on the FastMNN embedding. Correlation analysis of tdTomato^{+} and tdTomato^{-} cells within each γδ T cell cluster was performed based on principal component analysis embeddings. Then R package pheatmap was used to visualize the correlation efficient, and the hierarchical clustering method was set as "ward.D2".

PMA/ionomycin stimulation assay

Liver mononuclear cells were isolated from 2-month-old mice via Percoll density gradient centrifugation, and live CD45^{+}CD3^{+}TCRγδ^{+} γδ T cells were sorted using a FACS Aria III cell sorter (BD Biosciences). The sorted γδ T cells were stimulated for 4 h at 37 °C in RPMI 1640 medium supplemented with 10% fetal bovine serum (FBS) and Cell Stimulation Cocktail (Thermo Fisher Scientific, 00-4975-93), containing phorbol 12-myristate 13-acetate (PMA),

ionomycin, brefeldin A, and monensin. Following surface marker staining, cells were fixed, permeabilized, and stained intracellularly with monoclonal antibodies against IFN-γ and IL-17.

References

Haghverdi, L., Lun, A.T.L., Morgan, M.D., and Marioni, J.C. (2018). Batch effects in single-cell RNA-sequencing data are corrected by matching mutual nearest neighbors. Nat Biotechnol *36*, 421-427.

Hou, S., Li, Z., Dong, J., Gao, Y., Chang, Z., Ding, X., Li, S., Li, Y., Zeng, Y., Xin, Q.*, et al.* (2022). Heterogeneity in endothelial cells and widespread venous arterialization during early vascular development in mammals. Cell Res *32*, 333-348.

Liu, Z., Gu, Y., Shin, A., Zhang, S., and Ginhoux, F. (2020). Analysis of Myeloid Cells in Mouse Tissues with Flow Cytometry. STAR Protoc *1*, 100029.

Madisen, L., Zwingman, T.A., Sunkin, S.M., Oh, S.W., Zariwala, H.A., Gu, H., Ng, L.L., Palmiter, R.D., Hawrylycz, M.J., Jones, A.R.*, et al.* (2010). A robust and high-throughput Cre reporting and characterization system for the whole mouse brain. Nat Neurosci *13*, 133-140.

McGinnis, C.S., Murrow, L.M., and Gartner, Z.J. (2019). DoubletFinder: Doublet Detection in Single-Cell RNA Sequencing Data Using Artificial Nearest Neighbors. Cell Syst *8*, 329-337 e324.

Tang, W., He, J., Huang, T., Bai, Z., Wang, C., Wang, H., Yang, R., Ni, Y., Hou, J., Wang, J.*, et al.* (2021). Hlf Expression Marks Early Emergence of Hematopoietic Stem Cell Precursors With Adult Repopulating Potential and Fate. Front Cell Dev Biol *9*, 728057.

Supplemental table legends

Supplemental Table 1. Differentially expressed genes of hepatic cells.

Differentially expressed genes among all hepatic cells **(Fig. 4C)**. Statistical significance was determined by Wilcoxon rank-sum test with Bonferroni correction (adj. $p < 0.05$).

Supplemental Table 2. DEGs of hepatic γδ T cell subsets.

Differentially expressed genes among six γδ T cell subclusters (gdT_Sell, gdT_Il4, gdT_Ncr1, gdT_Gzmk, gdT_Rorc, gdT_Prolif) from mouse liver **(Fig. 4F)**. Statistical significance was determined by Wilcoxon rank-sum test with Bonferroni correction (adj. $p < 0.05$).

Supplemental Table 3. DEGs of naïve γδ T cells from different sources.

Differentially expressed genes among thymus, hepatic $tdTomato^-$ and hepatic $tdTomato^+$ naïve γδ T cells **(Fig. 5G)**. Statistical significance was determined by Wilcoxon rank-sum test with Bonferroni correction (adj. $p < 0.05$).
